# Epidermolysis Bullosa: Two rare case reports of *COL7A1* and EBS-GEN SEV *KRT14* variants with review of literature

**DOI:** 10.1186/s12887-024-04715-0

**Published:** 2024-04-05

**Authors:** Fatma Mabrouk Ali, Jieyu Zhou, Mingyan Wang, Qiuxia Wang, Lulu Sun, Mansour Maulid Mshenga, Hongyan Lu

**Affiliations:** 1 Department of Pediatrics, The Affiliated Hospital of Jiangsu University, Zhenjiang, Jiangsu China; 2 School of Public Health, Southern Medical University, Guangzhou, Guangdong China

**Keywords:** Epidermolysis bullosa, Blisters, Genetic testing, Collagen 7A1, Keratin 14

## Abstract

**Epidermolysis:**

Bullosa is a rare hereditary skin condition that causes blisters. Genes encoding structural proteins at or near the dermal-epidermal junction are mutated recessively or dominantly, and this is the primary cause of EB. Herein, two Chinese boys were diagnosed with the condition, each with a different variant in a gene that serves as a reference for EB genetic counseling. Skincare significantly impacted their prognosis and quality of life.

**Case presentation:**

Two Chinese boys, with phenotypically normal parents, have been diagnosed with distinct blister symptoms, one with Dominant Dystrophic Epidermolysis Bullosa and the other with a severe form of Epidermolysis Bullosa Simplex. The first patient had a G-to-A variant in the *COL7A1* allele, at nucleotide position 6163 which was named “*G2055A*”. The proband is heterozygous for Dystrophic Epidermolysis Bullosa due to a *COL7A1* allele with a glycine substitution at the triple helix domain. A similar variant has been discovered in his mother, indicating its potential transmission to future generations. Another patient had severe Epidermolysis Bullosa Simplex with a rare *c.377T > A * variant resulting in substitution of amino acid *p.Leu126Arg (NM_000526.5 (c.377T > G, p.Leu126Arg)* in the *Keratin 14* gene. In prior literature, Keratin 14 has been associated with an excellent prognosis. However, our patient with this infrequent variant tragically died from sepsis at 21 days old. There has been a reported occurrence of the variant only once.

**Conclusion:**

Our study reveals that Epidermolysis Bullosa patients with *COL7A1 c.6163G > A* and *KRT14 c.377T>A* variants have different clinical presentations, with dominant forms of Dystrophic EB having milder phenotypes than recessive ones. Thus, the better prognosis in the *c.6163G > A* patient. Furthermore,* c.377T>A* patient was more prone to infection than the patient with *c.6163G>A* gene variant. Genetic testing is crucial for identifying the specific variant responsible and improving treatment options.

## Background

Epidermolysis Bullosa (EB) is a group of heritable skin disorders typified by blister development resulting from structural fragility of the skin and other epithelial tissues under mild mechanical trauma [1].

The term EB was initially described in 1886, although the first satisfactory classification method was proposed by Pearson in 1962 with global prevalence rates of 1 out of 50,000 to 500,000 live births [2].

Currently, it is divided into four primary categories based on clinical and genetic characteristics namely (a) Epidermolysis bullosa simplex (EBS) (b) Junctional epidermolysis bullosa (JEB) (c) Dystrophic epidermolysis bullosa (DEB) and (d) Kindler epidermolysis bullosa [3]. The primary distinction between the four categories is the degree of skin separation and the production of blisters that follow [4].

EB can be mild blistering to life-threatening forms with clinical signs and symptoms ranging from localized to widespread blisters and skin sores throughout the body and oral cavity caused by secondary deficiencies [5]. These may complicate chronic wounds, scars, contractures, deformities, infections, or even problems affecting several internal organs such as the esophagus, the gastrointestinal and vesico-urinary tracts, as well as the extremities. This is brought by variants in genes that affect several skin protein types, including keratins and laminin 332 type VII collagen which in turn causes the skin to become fragile. So far, these variants have been identified in 16 genes, indicating the genetic diversity of EB [4, 6]. Type VII collagen (COLVII), an essential component of the anchoring fibrils, is encoded by the COLVII-A1 (Collagen-VII-A1) gene. Variants in this gene are the primary cause of the condition: epidermal keratinocytes and papillary dermal fibroblasts in the skin produce and deposit COLVII. We hereby present two cases, one with DDEB who had blisters on bilateral lower limbs and another with EBS-gen sev who presents with severe widespread blistering and nail absence at birth. We delve into how our team managed the infants’ disease course by treating the skin symptoms efficiently. Furthermore, we explore how early diagnosis through genetic testing can aid in accuracy and better outcomes.

## Case series presentation

### Case 1

**Fig. 1:**
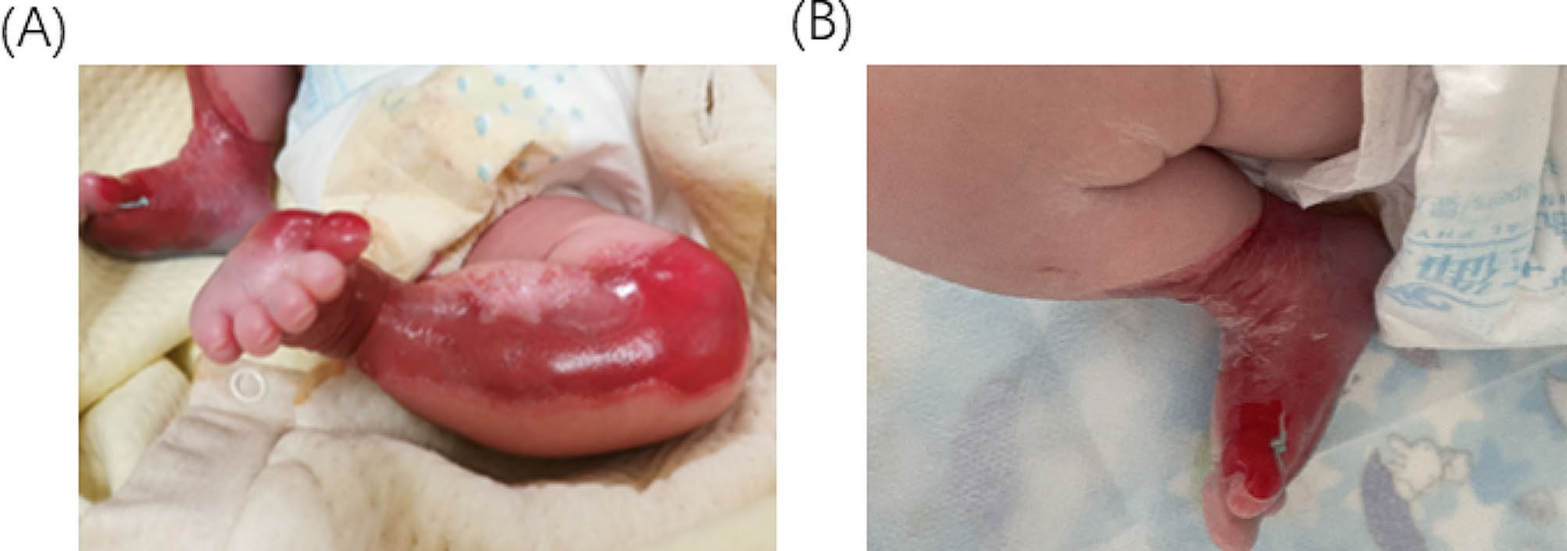
(**A**) Skin condition of the left leg on the first day of birth. (**B**) Skin condition of the right leg on the first day of birth

A 3-hour full-term male baby was referred to our hospital with bilateral asymmetrical ulcers. Despite weighing 3.1 kg and being born at full term via routine delivery without any issues, the baby presented with dermatological abnormalities that covered over 8% of his body surface and were mostly localized around both legs. The left, an irregular “S” shaped defect involves the medial aspect of the thumb, plantar region, extending up to above the knee measuring about 15*10cm **(**Fig. 1A). The right was a C-shaped defect around the calf area covering approximately 6 by 10 cm **(**Fig. 1B**).** Easily visualized vascular structures, a thin translucent membrane covering a bright red surface, and distinct separation from the normal skin were observed. All reflex tests presented normal except for bilateral lower limbs. The unmarried 32-year-old mother had an unremarkable medical history with no complications during the whole course of her pregnancy and denies any history of chronic or genetic disease in the family. Clinical evaluations revealed a healthy baby with no other anomalies.

**Fig. 2:**
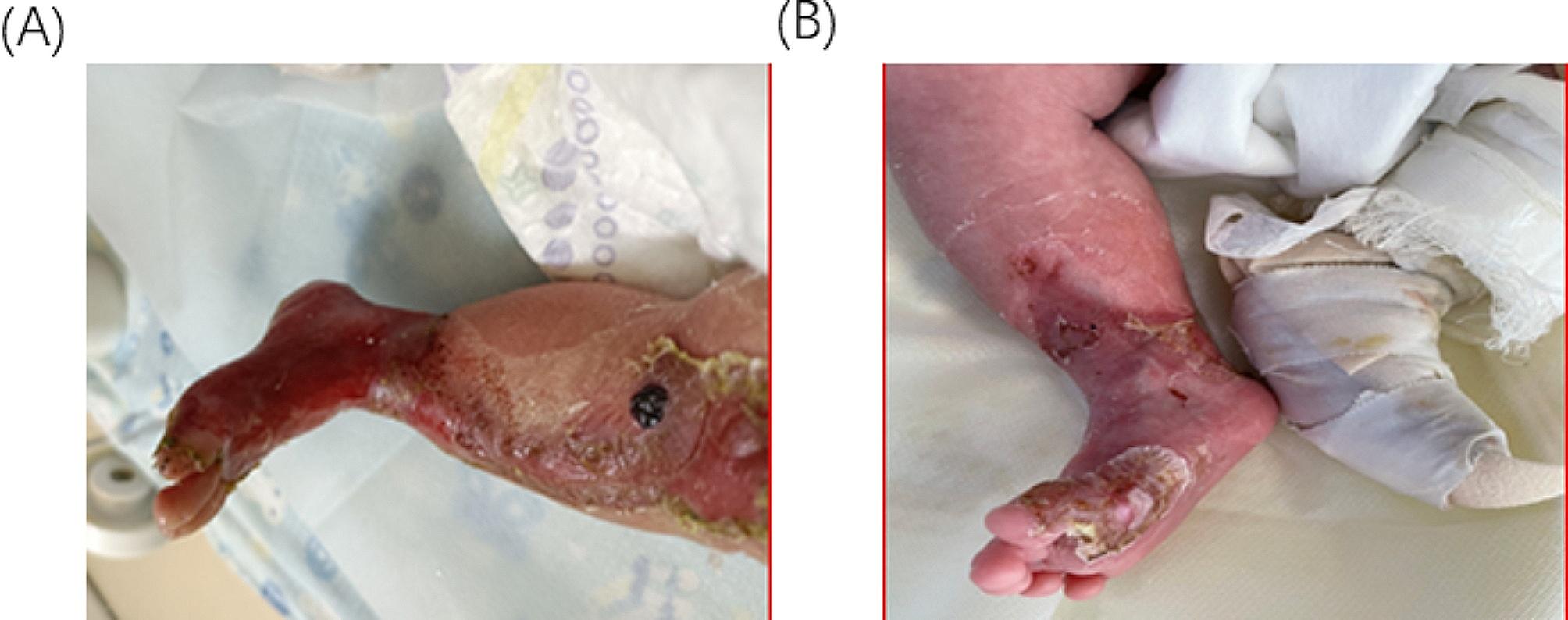
(**A**) 16 days after birth Left leg at 5th dressing change. (**B**) 16 days after birth Right leg at 5th dressing change

**Fig. 3:**
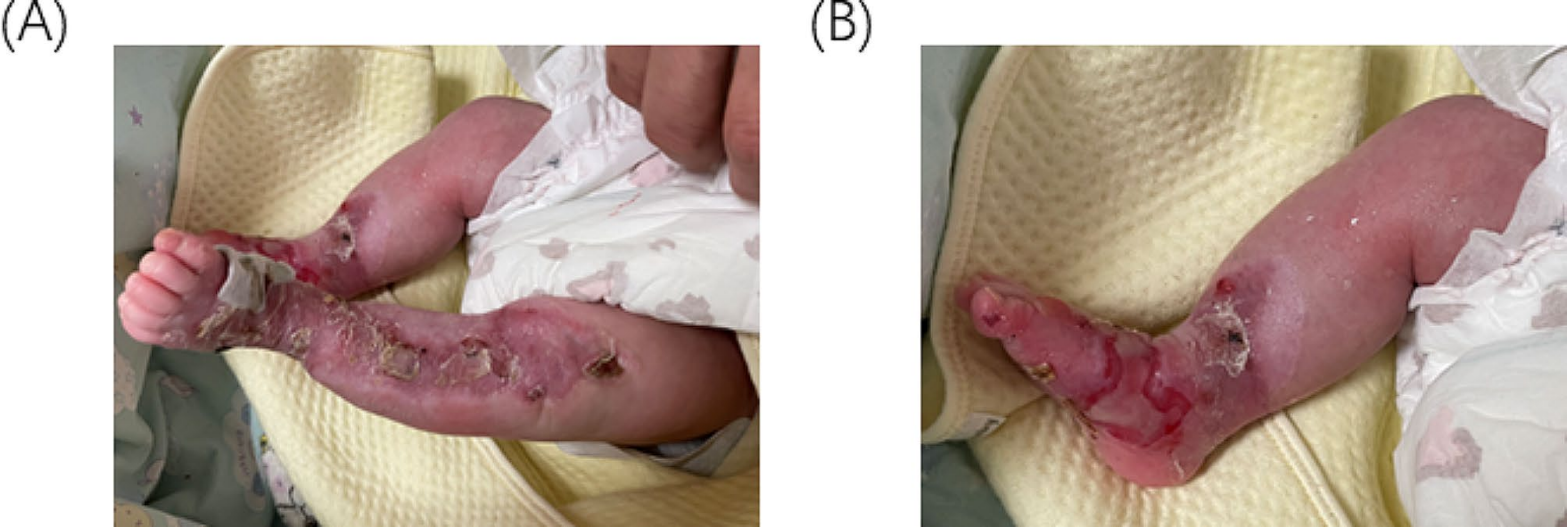
(**A**) 30 days after birth Left leg at 9th dressing change. (**B**) 30 days after birth Right leg at 9th dressing change

**Fig. 4:**
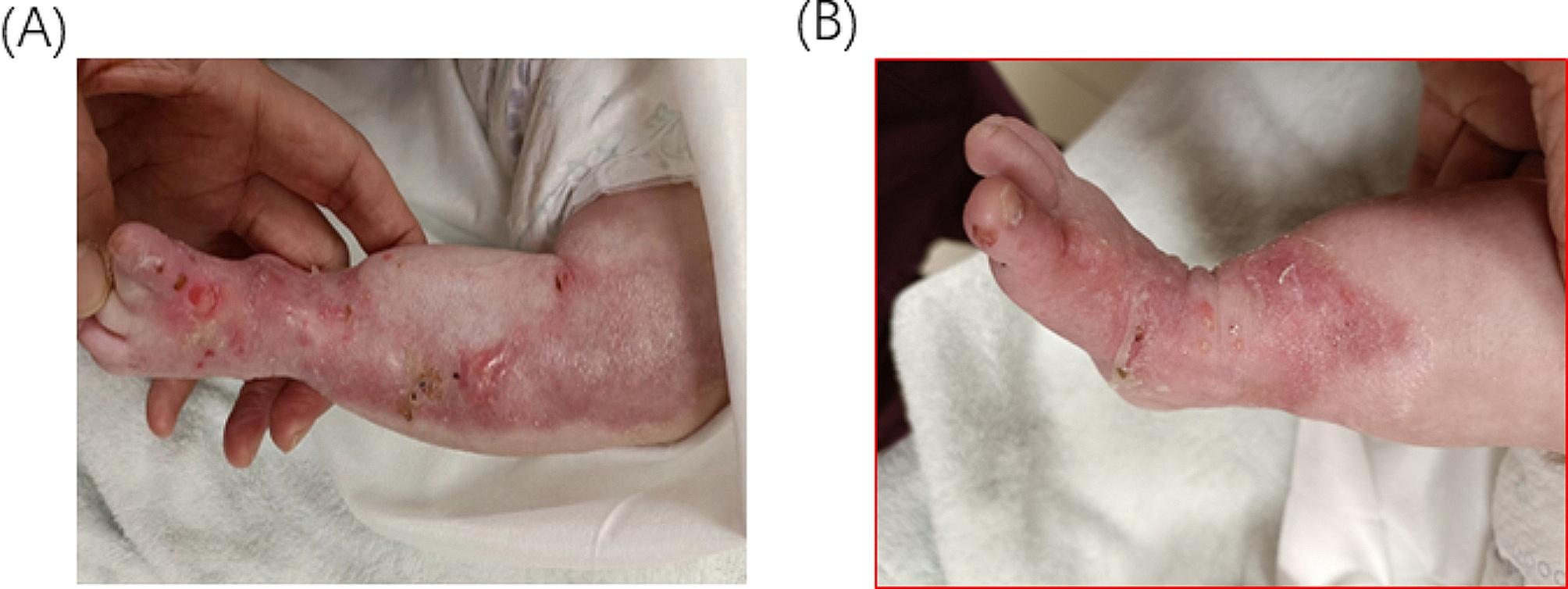
(**A**) 42 days after birth the 11th dressing is changed on the left leg before discharge. (**B**) 42 days after birth the 11th dressing is changed on the right leg before discharge

Our hospital management involved hydration support, and the use of prophylactic antibiotics, to prevent and treat infections. Non-adhesive polysilicon wound dressing was done in intervals of 3–5 days and using sterile water, and lysosome was useful in ensuring infection was controlled. The healing process started at the peripherals **(**Fig. 2, and 3**)**, and complete healing was attained in the following 45 days **(**Fig. 4A and B**).** The skin around the medial malleolus and the anterior part of the ankle joint were the final areas to recover.

**Fig. 5:**
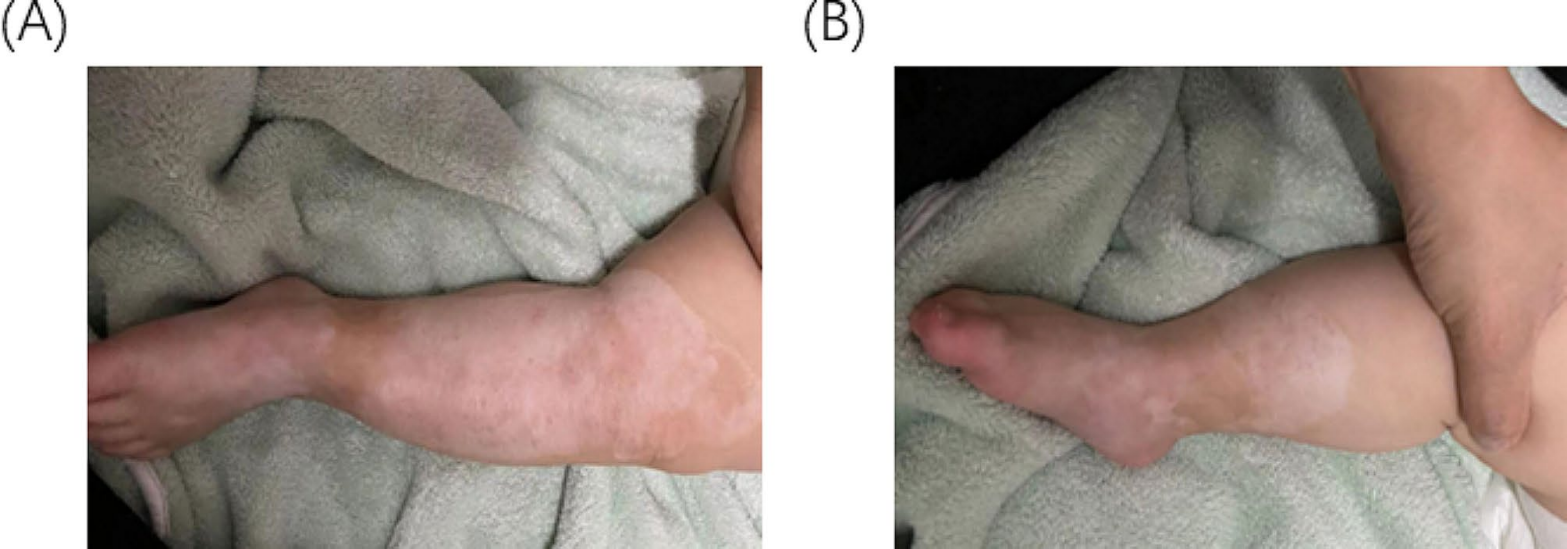
(**A**) Skin condition of the left leg at the age of 1 year. (**B**) Skin condition of the right leg at 1 year of age

**Fig. 6:**
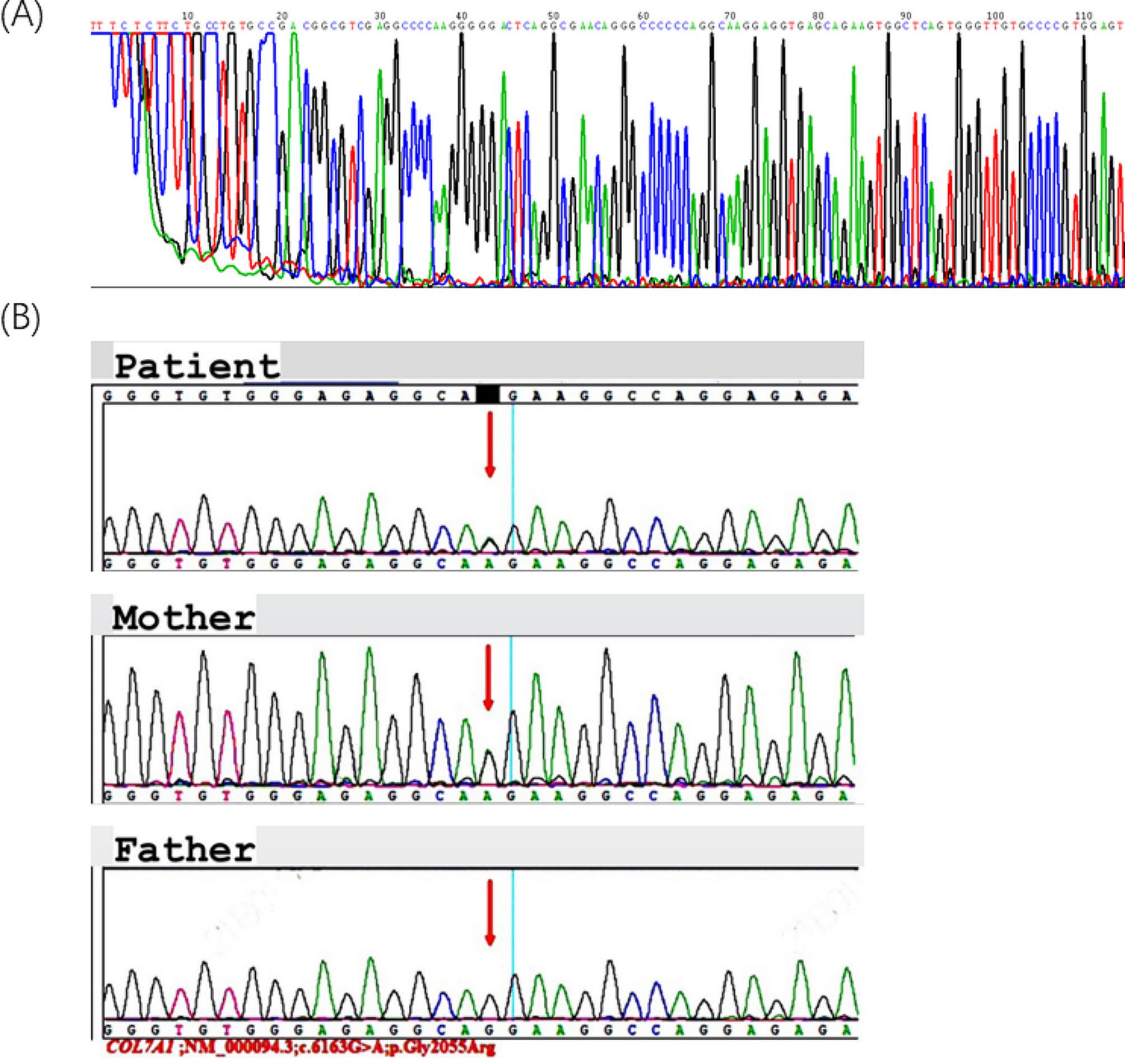
(**A**) Shows the patient’s Sanger sequencing technology revealing the *COL7A1* gene. b illustrates the patient’s and parents’ results from genetic testing showing the *COL7A1* gene. (**B**) A nucleotide change *(c.6163G > A*) in axon 73 and heterozygous variant in the *COL7A1(NM_000094.3)* gene was present in the affected individuals. Additionally, there is an amino acid change *(p. Gly2055Arg*). This genetic variation is situated at location 48,612,789 on chromosome three, in the EX73/CDS73 region. Red arrows indicated nucleotides that were mutated as seen in the Figure

With informed parental consent for genetic testing pertinent to their child’s health status, the perilesional skin was mapped using immunofluorescence antigen during the first week of life. The results revealed a link between pathogenic variant; *chr3: 48,612,789; c.6163G > A; p. Gly2055Arg in EX73/CDS73* generating an EB diagnosis and an autosomal dominant inheritance pattern whereby the child inherited this gene from the carrier mother (Fig. 6). Following a year, the baby underwent follow-up testing to look for any anomalies or developmental issues **(**Fig. 5A and B**)**.

Specific blood tests, standard stool tests, and bacterial smear tests were performed. No problems or abnormalities were found during the testing process, indicating that all levels were within the anticipated normal ranges.

Additionally, **(**Fig. 6A, and 6B) showed first-generation sequence and genetic results of the infant with heterozygous variations in the *COL7A1* gene that might connect the development of Aplasia Cutis Congenita with chromosome 3 alterations. Sanger sequencing is widely used as the first step in genetic testing because of its effectiveness in identifying gene alterations and providing accurate diagnoses.

## Case 2

**Fig. 7:**
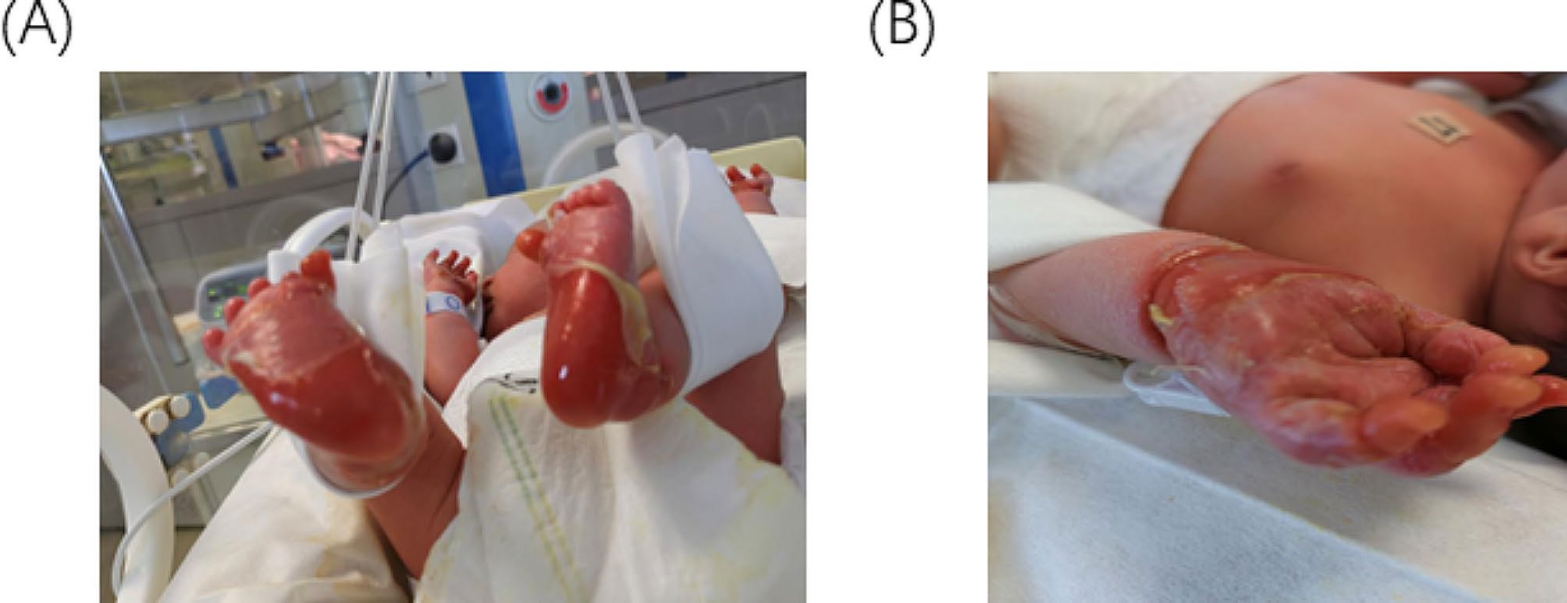
(**A**) On the first day of life, the baby’s feet feature a brilliant red, skinless region. (**B**) a bright red, skinless region covers the baby’s hands

**Fig. 8:**
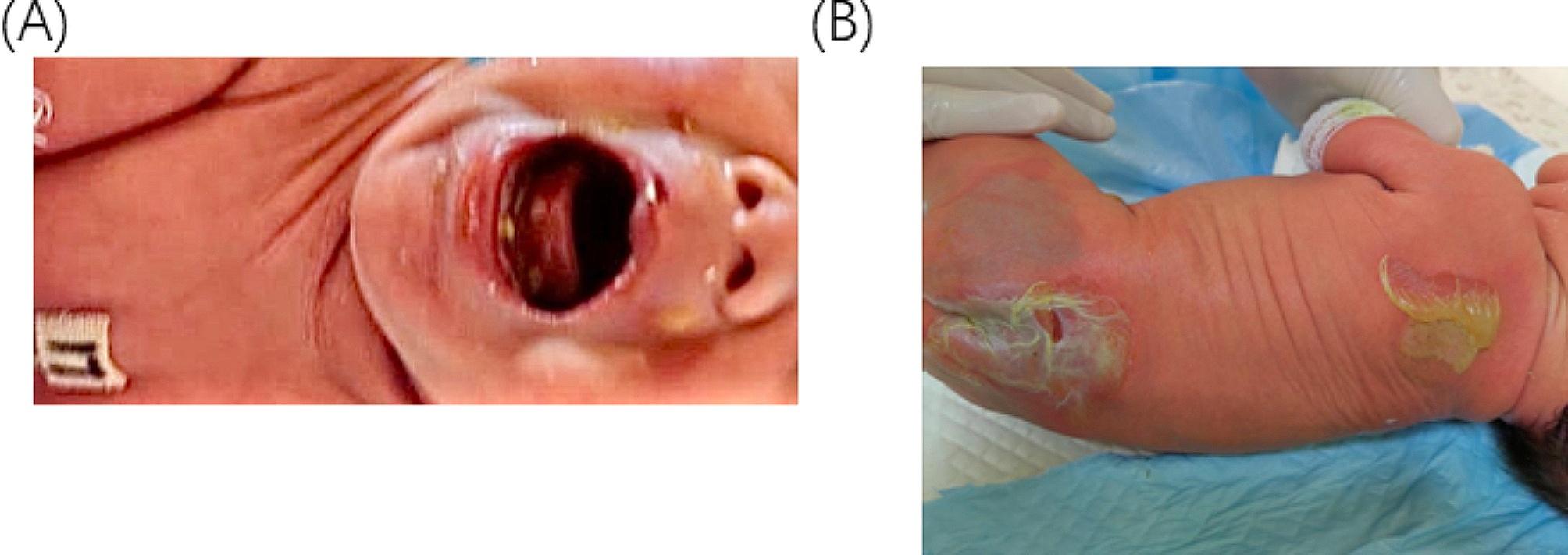
(**A**) on day 3 illustrates a mucosal erosion that started in the mouth and spread to the nose’s wings. (**B**) displays a visible, exfoliated bolus covered in exudate on the buttocks upon birth

2h after birth, a spontaneously delivered male baby was brought to Jiangsu University Hospital following the discovery of a shinny red area with no skin on the hands, or feet **(**Fig. 7A and B**)** and an exfoliated epithelial-covered bolus on the buttocks with visible exudate and no purulent discharge (Fig. 8B). The child also presented with a congenital absence of fingernails and toenails. New blisters began to form from the skin defect’s margins to the medial portion. He suffered a mucosal erosion first on his mouth, which later spread to the wings of his nose (Fig. 8A). Extremities have normal muscle tone and primitive reflexes elicitation.

Despite being delivered at the GA of 37 + 4-week, with an Apgar score of 9/10, and having no relevant history of this disease, postnatal illness, or any other chronic illness, the patient had multiple blisters and erosions throughout the first few days of life, with a preference for places that were mechanically exposed. However, other than these abnormalities, his vital signs and general health, including weight, length, and head circumference, were normal. The 31-year-old mother denied having taken any medications or being exposed to radiation while she was pregnant. No skin anomalies or mucous membranes were visible in either parent, who appeared to be in good health. The baby is the third child of this unmarried family, the first is alive but suffers from sensory disorders and autism. However, she had a miscarriage in her second pregnancy due to unknown causes. Using serology and microbiological cultures to screen for inflammatory, autoimmune, metabolic, viral, or microbial causes of newborn blistering revealed no pathology. The findings of the liver function test, renal function test, and complete blood count were all normal. Both a cardiac echocardiogram and an abdominal ultrasound showed no abnormality. To prevent further skin damage we changed positions regularly, used non-adherent wound dressing, kept him on IV antibiotics, vitamin K to prevent bleeding, started him with nasogastric tube feeding and fluid replacement support. Gamma globulin was also administered to modulate immunity due to the elevated risk of secondary infection.

But subsequently, the baby started to deteriorate, he had hyperrespiration with oxygen saturation ranging between 90 and 94%. In addition, he developed a fever, which peaked at 37.9 to 39.6 degrees Celsius. A chest x-ray was obtained; the findings suggested pneumonia because of the small patches and bilateral lung consolidation. Based on elevated WBC and CRP levels, Table 1 validates the diagnosis of neonatal sepsis.

**Table 1:** The results of the investigations done in the two patients

PATIENT’ INVESTIGATION RESULTS	NORMAL RANGE	RESULTS ON ADMISSION		RESULTS BEFORE DISCHARGE/ DEMISE	
		**COL7A1**	**KRT14**	**COL7A1**	**KRT14**
C-REACTIV PROTEIN	0-10mh/L	< 0.5 mg/L	< 0.5 mg/L	< 0.5 mg/L	184.8 mg/L
WHITE BLOOD CELL	3.5–9.5 10^9/L	10.0 10^9/L	5.4 10^9/L	6.6 10^9/L	12.3
HEMOGLOBIN	140–180 g/L	189 g/L	210 g/L	114 g/L	115 g/L
PLATELET COUNT	125–350 10^9/L	269 10^9/L	282 10^9/L	316 10^9/L	408 10^9/L
INTERNATIONAL NORMALIZED RATIO	0.8–1.2 s	1.2	1.2	-	-
PLASMA PROTHROMBIN TIME	9–13 s	13 s	14.00 s	-	-
PARTIAL THROMBOPLASTIN TIME	23.3–32.5	38.6	59.40 s	-	-
D- DIMER	< 0.55 mg/L	4.17 mg/L	2.43 mg/L	-	-
CULTURE AND SENSITIVITY.	Negative	Negative	Negative	Negative	Negative
STOOL FOR OCCULT BLOOD.	Negative	Negative	Negative	Negative	Negative

Abbreviations: s: time in seconds; -: no available results

**Fig. 9:**
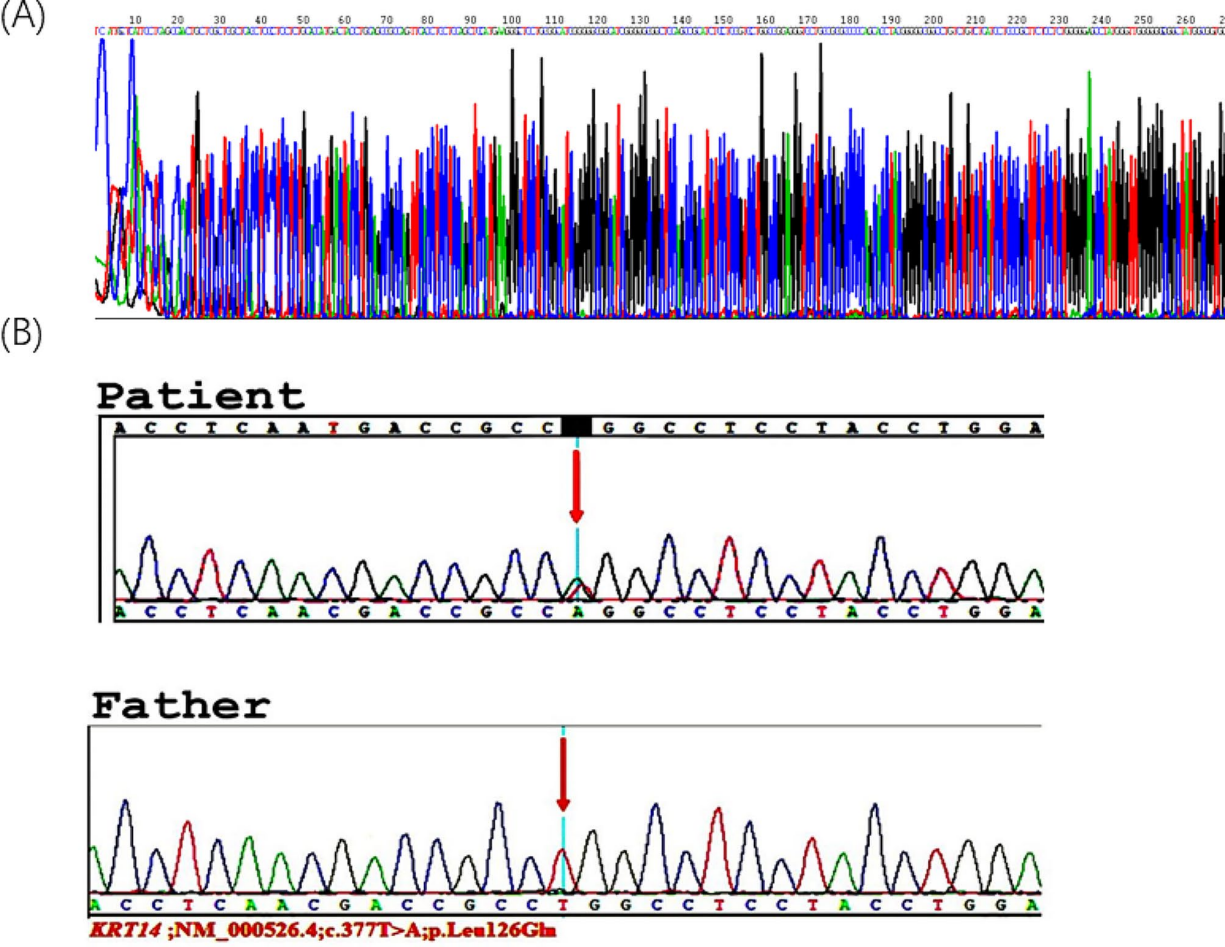
(**A**) Sanger sequencing of the patient’s genome revealed the *KRT14* gene. (**B**) The patient’s and his father’s genetic testing outcomes, displaying the *KRT14* gene. NM_000526.4 is the reference sequence that illustrates the nucleotide change *c.377T > A* and the amino acid changes *p.Leu126Gln* at the gene subregion EX1/CDS1 with chromosomal location chr17:39742710; was detected in the patient but was absent in the patient’s father

Consequently, we changed the baby’s medication to meropenem and kept the child on Oxygen, but the condition did not improve. Sadly, the infant died of sepsis after 21 days of hospitalization. The results of the genetic test revealed a unique variant in *KRT14*, known to cause EBS, *c.377T > A*, resulting in the amino acid substitution *p.Leu126Gln* on chromosome 17, consistent with the child’s symptoms. The mother of the child did not perform any genetic testing due to economic reasons, his father, however, displayed no *KRT14* variant in his results (Fig. 9B). As a result, the diagnosis was established based on the clinical evaluation, the child’s, and his father’s immunofluorescence results. This patient experienced recurrent episodes of extensive bullous growth and did not considerably thrive. In the first several weeks of his life, he experienced multiple infections and poor weight gain. The most potent antibiotics were not able to improve the condition of the patient.

The father received genetic counseling, which explained the significance of these variants and recommended suitable solutions for the Child’s prognosis. (Figure 9A and B**)** displays the first-generation sequence and genetic testing result of the parents and patient of the child with *KRT14* variant respectively.

## Discussion

Two patients with severe forms of heterozygous hereditary EB were identified; the first patient’s clinical presentation, which comprised skin absence with bullae in both lower extremities, led to the diagnosis of both ACC and DDEB. The three most prevalent forms of DEB are “RDEB, generalized intermediate,” “DDEB, generalized,” and “RDEB, generalized severe.” “DDEB, generalized” has a favorable prognosis and exhibits decreased collagen VII expression. Blisters are localized to the site of damage and are minor. Rarely do teeth and the oral mucosa become involved [7]. The amount of disruption in Dystrophic Epidermolysis Bullosa occurs at the level of the anchoring fibrils, below the lamina densa, and causes the blisters.

**Fig. 10:**
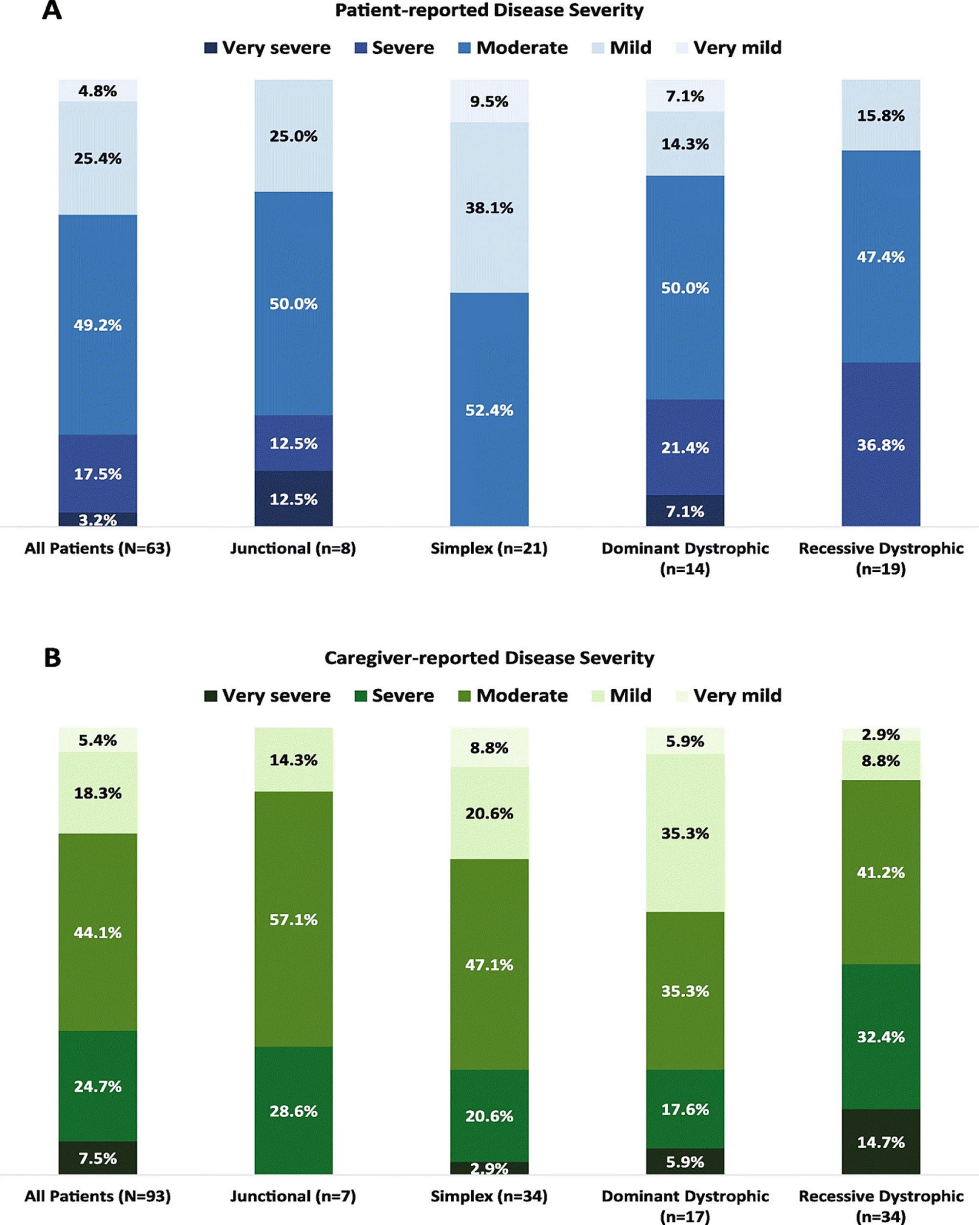
A comprehending the severity of epidermolysis bullosa via patient and carers’ feedback

**Fig. 11:**
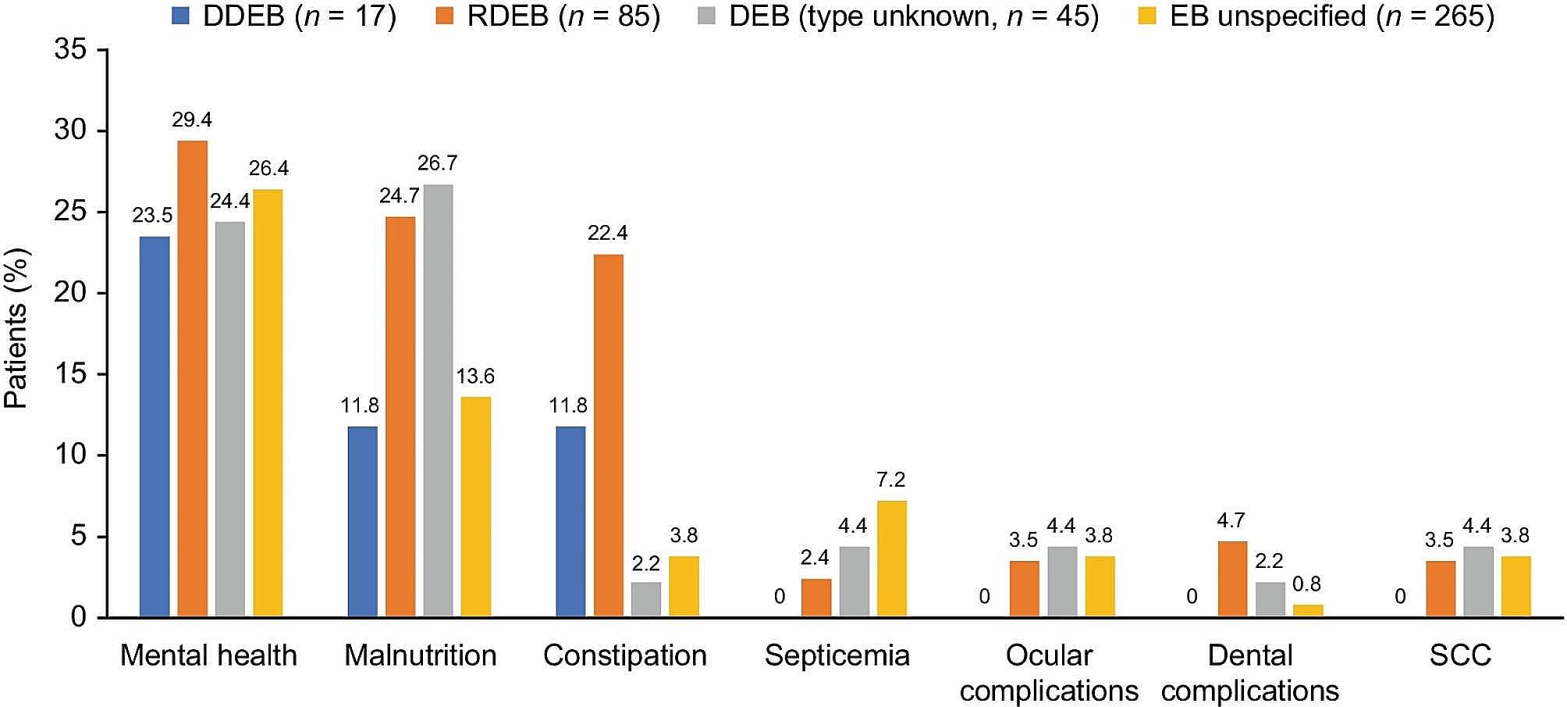
Comorbidities for Patients with SCC, RDEB, EB, and DEB at a 12-Month Follow-Up

The genetic test results of the our patient showed that the infant inherited the heterozygous *COL7A1 c.6163G > A *variant from the carrier mother, validated the diagnosis. A nucleotide substitution caused by the variant “*G2055A*” changed a glycine codon (GGA) into a glutamic acid codon (GAA). Although there are some existing reports of this variant from prior literature [8], new variants are still being discovered [9]. According to Diociaiuti and Randhir’s findings (Table 2), *COL7A1* variants have been found to represent a dominant inheritance pattern in several cases of lower extremity aplasia cutis congenital and Bollus Dermolysis of the Newborn (BDN) [8, 10]. Our case findings, however, differ from Valeria Venti’s case, which identified a novel homozygous single-base missense variant *c.6797G > T* in exon 86 of the COL7A1 gene which had not been reported prior on the DEB registry [8, 11, 12]. A few variants in the *COL7A1* gene are recurrent but many variants are exclusive to specific families. *G2055A*, found in our patient, differs from other variants, such as *5818delC, 6573 + 1G–> C*, and *E2857X*, found in Japanese and British populations. Some studies suggest that *G2043R* and *425 A–> G* variants are a global hotspot. Seven out of ten patients with the *G2043R *variant have it occurring de novo. Screening for ethnically specific variants and *425 A-G* may be effective in *COL7A1* variants searching [13–15]. Other studies indicate that in patients who are suspected of having DDEB, sequencing analysis of exons 73–75 of the *COL7A1* gene can identify about 75% of the causal variants. (as well as 95% of instances if a precise DEB diagnosis is made [16]. Our patient’s better prognosis can be attributed to dominant variations of dystrophic EB, which are milder and less severe than recessive forms, according to examples previously reported in the literature. A Bruckner study found that patients with recessive dystrophic epidermolysis bullosa had the highest severity rating, with 75% classifying it as moderate to very severe (Fig. 10A and B). Further studies found frequent complications, including constipation and malnourishment having individuals with RDEB experiencing more complications than DDEB **(**Fig. 11) [17, 18].

**Table 2:** The several literatures of instances with various subtypes of epidermolysis bullosa

AUTHORS	PATIENTS’ AGE AT SYMPTOM ONSET	CLINICAL PRESENTATION	GENETIC TESTING RESULTS	TREATMENT STRATEGY
Randhir Sagar Yadav^1^ 2018	Diagnosed at 26y (SONS)	A variety of vesicles and bullae with hemorrhagic crusts and erosions	Dominant Dystrophic EB COL7A1	Symptomatic treatment with mupirocin ointment and paraffin gauze.
Andrea Diociaiuti ^2^ 2016	2 days old.	The presence of hemorrhagic blisters.	COL7A1	NM
Jing Liu^3^ 2021	At birth.	Absence of skin on the lips, face, and both lower limbs.	COL7A1	Antibiotics administered, aseptic dressing changes.
Kotaro Komatsu^4^ 2020	At birth throughout infancy.	Generalized recurrent. Severe pruritis with blisters. And mild hypoplasia of the toenail. Currently Presented with atrophic scarring.	DDEB, p. G2043R is the heterozygous single nucleotide alteration c.6127G > A in exon 73 of COL7A1, arginine residue.	NM
Christine Cramer ^5^ 2018	At birth.	Blistering and skin erosions	ACC with Recessive dystrophic EB	Antibiotic treatment, analgesics, non-adherent silver nitrate dressings, and cotton gloves to prevent adhesions.
Valeria Venti ^6^ 2020	During childhood (SONS).	Skin erosions and scarring since childhood with blisters evolving into painful ulcers.	*COL7A1* gene revealed a homozygous single-base missense variant c.6797G > T.	Intravenous fluid, antibiotic therapy, oral iron supplementation, topical agents, and non-adhesive dressings.
Jia Zhang ^7^. 2016	Since birth.	Generalized blisters, worsened by friction absent nails, and hyperkeratosis.	KRT5- EBS-gen-sev	Minimizing trauma, good nutrition, and infection control
Sunitha Tella ^8^. 2022	Presented at 2y (SONS)	Blisters, scars, hypopigmented areas, and dystrophic nails.	Autosomal Recessive- EBS with nail and muscular dystrophy. PLEC gene (chr8:144998220delC; c. 6288del; p. Arg2097AlafsTer55) and (chr8:145001693_145001694delCT; c.4054_4055del; p.Ser1352CysfsTer68)	NM
Mohammed Al Towijry ^9^ 2023	3 months old.	Recurrent vesicles and bullae on non-pressure sites of hands and feet. Teeth abnormality and caries.	Homozygous for Dystonin (EBS gene mutation) c.3370 C > T, p. (Gln1124)	Supportive, TA, nutritional support, and preventive measures of blister formation.
Jana Kyrova^10^ 2016	8 years old.	Skin and oral mucosa erosions, hemorrhagic blisters, onychodystrophy, and later severe scoliosis.	EBS- with Muscular Dystrophy. PLEC gene mutation.	NM
Aleksandra Bergant Suhodolčan ^11^ 2014	Soon after birth.	Blisters, crusts, erosions around the navel, and blisters on fingers and toes.	EBS with mottled pigmentation KRT5:c.74 C > T variant (missense KRT5:p.Pro25Leu mutation)	TA, prevention of secondary infection and trauma.

Abbreviations: SONS - Symptom onset not specified; y - age in years; NM - Not Mentioned Mentioned; TA - Tropical antibiotics

The second patient’s diagnosis in our case series is EBS with *c.377T > G* variant, amino acid substitution *p.Leu126Arg (NM_000526.5 (c.377T > G, p.Leu126Arg*) on chromosome 17, which is known to cause EBS, who presented with generalized blisters and even mucosal involvement. Three main forms of EBS exist: localized (EBS-loc; OMIM number. 131,800), generalized severe (EBS-gen sev; OMIM no. 131,760), and generalized intermediate (EBS-gen intermed; OMIM no. 131,900). EBS-gen-sev is the most severe form of EBS and is caused by a variant in the keratin 5 *KRT5 or KRT14*. Autosomal recessive EBS is associated with both biallelic pathogenic variants in *CD151* (a member of the tetraspanin superfamily), *DST* (Dystonin), or *EXPH5* (exophilin-5) and biallelic loss-of-function variations in *KRT5, KRT14, or PLEC* (Plectin). Autosomal dominant EBS is associated with either a heterozygous pathogenic variant in *KLHL24* or a heterozygous dominant-negative variant in *KRT5, KRT14, or PLEC* [19]. .

In EBS, blisters form on the basal keratinocytes, however, other research revealed that the development of epidermolysis bullosa simplex blisters was caused by the disintegration of basal and suprabasal cells. Blisters can range in severity from only affecting the hands and feet to being all over the body [20]. Skin fragility in EBS leads to non-scarring blisters and erosions brought on by slight mechanical damage. In some cases, this fragility also affects mucosal epithelia. The location of the blistering concerning the dermal-epidermal junction serves as its defining characteristic, which is distinguished from other kinds of EB or non-EB skin fragility syndromes.

The case shares similarities with one discussed by Zhang et al. (Table 2), except for congenital hyperkeratosis. Jana Kirova’s findings, discuss an EBS with muscular dystrophy (EBS-MD) along with hemorrhagic blistering, different from our patient’s [21, 22]. Autosomal dominant inheritance accounts for most cases, however, rare autosomal recessive types of EBS have been documented [23–26]. A high rate of 37% for de novo pathogenic variants in *KRT14* and *KRT5* has been observed in a separate study. Highly changeable CpG dinucleotides have been discovered in several codons that are more commonly impacted by these de novo variants in numerous families, however, the exact reason for the high frequency of de novo variants is yet unknown [27]. Since only one parent’s DNA was analyzed in our patient and the variant was discovered to be absent, we cannot definitively assume that this is a De novo variant. In the absence of a family history of EBS, the patient’s severe generalized EBS is explained by pathogenic variants in the *KRT14* genes.

To the best of our knowledge, the *KRT14* gene has been associated with multiple cases with a range of several variants in the literature, all of which have demonstrated a rather decent prognosis. On the contrary, there is just one known case of variant *c.377T > A*, which is the same variant our patient had, and that patient likewise had a severe form of EBS [28]. Numerous academic publications have documented sepsis-related deaths in *KRT14* patients; for instance, like our patient, another EBS instance was described in which the patient also experienced septicemia [29]. However, our patient’s *c.377T > A* variant appears to make patients much more vulnerable to infections [30–32]. Sepsis, a complication associated with EB, is prevalent in various incidences, with septicemia being the most common in unspecified types of EB (Fig. 11), including the EB simplex type. In all forms of EB, oral erosions and blisters are incredibly common. Like our patient who has oral erosions and ulcers (Fig. 8a), their involvement results in neuropathic pain which makes it difficult to feed and thus, the increased likelihood of malnutrition and inability to thrive [33–35]. Long recognized, some kinds and subtypes of hereditary EB may be susceptible to one or more extracutaneous complications, based on case reports and small case studies seen previously. Many of them have a significant risk of morbidity, and some can even be fatal, such as in our *KRT14* patients. Due to the complexity of the categorization and the large number of clinical subtypes of EB, an accurate classification of EB subtypes based only on clinical presentation is challenging. EBS-gen sev is the most severe form of EBS. It is caused by variants in the keratin 5 (*KRT5*) or *KRT14* genes which pose a life-threatening risk to newborns. However, after infancy, particularly in late childhood and maturity, the prognosis improves. Furthermore, the prognosis for *KRT14* individuals is usually bleak, some may even show significant skin abnormalities. For instance, our patient’s hands and feet were susceptible to adhesions, scarring, and contractures that might potentially hinder normal function [36–38]. 

Our approach to treating both patients involved minimizing skin fragilities by lowering the risk of secondary infections and making sure there were as few stressful situations as possible, even if conservative treatment has shown favorable results in a few cases. Prophylactic antibiotic use can lower infection risk and speed up wound healing, especially in patients with ACC and EB, as further evidenced by McCarthy and Simman et al. [39, 40]. Several other cases have shown positive outcomes [41]. By far, very few controlled trials have been conducted for the treatment of EB [42–44]. However, in most cases, the mainstay of treatment consists of supportive measures such as wound care, infection control, nutritional assistance, and management of any complications that may arise [45]. To facilitate this, numerous guidelines about wound care, wound cleansing, wound healing, and support for practitioners and carers have been developed to enhance the well-being of these patients [46, 47]. It is essential to managing and preventing infections, however, this may cause excruciating pain. Therefore, the psychosocial clinical practice guidelines were developed, which focused on pain management and improved the quality of life [45]. It is recommended to manage EB using the emergency care protocols [48] and a multidisciplinary approach. Thus, a group of dietitians, dermatologists, and nurses may be consulted when indicated [49, 50], since one of the primary challenges these patients face is malnutrition, as indicated in Fig. 11.

Certain complications that might develop in severe cases of EB may require surgery to manage. For instance, thoracoscopic surgery was performed on an EBS patient who experienced pneumothorax, with outstanding results [51]. However, implementing gene replacement therapy in EB patients has recently been shown to be of great benefit to improving prognosis and restoring patient’s quality of life [52–54]. Interestingly, some researchers have found that DDEB patients’ leg inflammation can be significantly reduced by topical tacrolimus 0.03% medication. Nonetheless, some discovered that the patient’s symptoms and quality of life improved after receiving low-dose botulinum toxin treatment [55, 56].

### Clinical comparison

Due to differences in the variant sites, the two-case series of newborns with *c.6163G > A* variant and *c.377T > A * variants presented differently clinically. The *COL7A1 c.6163G > A* patient phenotype was less severe with the denuded skin only located on the lower limbs, while NGS analysis of the *KRT14* patient revealed a *c.377T > G* variant having more widespread blisters that affected the hands, feet, back, buttocks, nose, and oral mucosa, which gave more room for bacterial penetration, leading to unresolved septicemia and ultimately leading to his death.

Furthermore, unlike our *KRT14* patient, the *COL7A1* patient had Aplasia cutis Congenita and skin absence at birth, which is a defining marker of DDEB. *COL7A1* variant patients have blisters that heal with scars as seen in our patient in (Fig. 5A and B). Unlike the *KRT14* variant, which are non-scarring blisters.

Blisters in *COL7A1* are mostly prominent over acral sites. However, in *KRT14* variants, blisters can affect the hands, feet, knees, and elbows but may additionally lead to regions of the trunk progressively becoming interspersed with hypopigmented patches.

*COL7A1* induces dystrophic nails, particularly in the toenails; even though our patient’s nails were normal. *KRT14* dystrophic nails can occur in the hands or feet, assuming they even have nails at all. As demonstrated by our *KRT14* patient whose nails were absent.

Unlike our *COL7A1* patient, our *KRT14* patient had extensive palmar and plantar hyperkeratosis. While no copy number variants were found, NGS analysis of the *KRT14* patient revealed a heterozygous *c.377T > G* variant in the *KRT14* gene, which was predicted to result in the amino acid substitution *p.Leu126Arg (NM_000526.5 (c.377T > G, p.Leu126Arg).* Squamous Cell Carcinoma is a common complication of the *COL7A1* variant, unlike the *KRT14* variant where they are not commonly predisposed to this complication.

### Genotype-phenotype correlation

Due to distinct genetic variants and the difference in their respective variant locations, the two patients exhibited varying phenotypic presentations and prognoses. Several academic works discuss the phenotypic severity of recessive forms of DEB in comparison to dominant ones; our patient, however, had a dominant form of DEB, which accounts for his remarkable prognosis in contrast to the patient with *c.377T > G* variant. One of the main causes of death for patients with *KRT14* variants is sepsis; additionally, individuals who have *c.377T > A* variant, appear to be much more susceptible to infection than those with *c.6163G > A* variant.

### Importance of genetic testing

EB diagnosis requires a comprehensive medical record, clinical assessment, and genetic testing due to its lack of recognition. Genetic testing aids in identifying the best treatment. To promptly counsel families on the natural history of the disease, risk of recurrence, and reproductive alternatives, experts must be knowledgeable about inheritance, age-related morbidity, and mortality linked to EB. Histopathology and molecular research are crucial for prognostication and counseling. Using genetic testing, variants in EB-related genes can be directly detected, improving clinical diagnosis to find the disease’s cause and, ultimately, a patient’s prognosis. Genetic testing is particularly advised for infertile couples or those with a history of miscarriages or stillbirths, as it can yield more thorough analysis reports and identify new variants and paternal carriers directly. In early pregnancy, genetic testing can also be performed to screen for autosomal recessive malnutrition type EB, provide scientific fertility advice, and determine the likelihood that an offspring will carry the disease.

### Limitation

The main drawback of the study is that our patient’s mother, who carries the *KRT14* variation, did not consent to having her DNA examined. As a result, we were unable to entirely rule out the chance that she carried the variant.

There are a few numbers of cases in our study.

## Conclusion

The study reveals that dominant forms of dystrophic epidermolysis bullosa (DDEB) have milder phenotypes and are less severe than recessive forms. *KRT14* variants cause sepsis, and *c.377T > A* variants increase infection susceptibility than *c.6163G > A* variant. Genetic testing is crucial for identifying the variant responsible and improving treatment options. Monitoring body temperature, WBC count, and CRP daily can aid in early diagnosis of sepsis.  Our work contributes to the growing knowledge regarding *COL7A1* and *KRT14* variants.

## Data Availability

Data and materials of this case study are to be provided upon request through the corresponding author.

## Abbreviations

COL7A1: Collagen Type VII Alpha 1 Chain
KRT14: Keratin 14
KRT5: Keratin5
DDEB: Dominant dystrophic Epidermolysis Bullosa
RDEB: Recessive Dystrophic Epidermolysis B
DEB: Dystrophic Epidermolysis Bullosa
EBS: Epidermolysis Bullosa Simplex
JEB: Junctional Epidermolysis Bullosa
AD: Autosomal Dominant
AR: Autosomal Recessive
OMIM: Online Mendelian Inheritance in Man (OMIM)
CDS: coding sequence
EBS-gen-sev: Epidermolysis bullosa simplex generalized severe
EBS-loc: Epidermolysis Bullosa Simplex Localized
EBS-gen intermed: Epidermolysis Bullosa Simplex generalized intermediate
EBS-gen sev: Epidermolysis Bullosa Simplex generalized severe
PLEC: Plectin
DST: Dystonin
EXPH5: Exophilin-5
ERN-Skin: European Network for Rare Skin Disorders
